# Supplementary material for: Association of triglyceride glucose-body mass index and hemoglobin glycation index with heart failure prevalence in hypertensive populations: a study across different glucose metabolism status
Source: Lipids Health Dis. 2024 Feb 22;23:53. doi: 10.1186/s12944-024-02045-9 (PMC10882741; doi:10.1186/s12944-024-02045-9)
Supplement: Supplementary file 1 — Supplementary Material 1 [file 12944_2024_2045_MOESM1_ESM.docx]

| **Table S1.** **Best threshold analysis of TyG-BMI and HGI for the detection of HF** | | | | | | | |
| --- | --- | --- | --- | --- | --- | --- | --- |
| **Test** | **Best threshold** | **Specificity** | **Sensitivity** | **Accuracy** | **Positive-LR** | **Negative-LR** | **Diagnose-OR** |
| **Total** |  |  |  |  |  |  |  |
| TyG-BMI | 342.89 | 0.87 | 0.22 | 0.82 | 1.64 | 0.90 | 1.82 |
| HGI | 0.25 | 0.76 | 0.36 | 0.73 | 1.49 | 0.84 | 1.77 |
| **Normoglycemia** | |  |  |  |  |  |  |
| TyG-BMI | 194.27 | 0.81 | 0.29 | 0.79 | 1.53 | 0.88 | 1.75 |
| HGI | -0.19 | 0.40 | 0.70 | 0.41 | 1.17 | 0.76 | 1.54 |
| **Prediabetes** | |  |  |  |  |  |  |
| TyG-BMI | 326.08 | 0.83 | 0.23 | 0.79 | 1.37 | 0.92 | 1.49 |
| HGI | 0.47 | 0.88 | 0.20 | 0.83 | 1.62 | 0.91 | 1.78 |
| **Diabetes** |  |  |  |  |  |  |  |
| TyG-BMI | 342.88 | 0.74 | 0.38 | 0.70 | 1.48 | 0.83 | 1.78 |
| HGI | 0.41 | 0.59 | 0.48 | 0.58 | 1.16 | 0.89 | 1.31 |

Abbreviations: TyG-BMI, triglyceride glucose-body mass index; HGI, hemoglobin glycation index; HF, heart failure; LR, likelihood ratio; OR, odds ratio.
