# Supplementary material for: Association of triglyceride glucose-body mass index and hemoglobin glycation index with heart failure prevalence in hypertensive populations: a study across different glucose metabolism status
Source: Lipids Health Dis. 2024 Feb 22;23:53. doi: 10.1186/s12944-024-02045-9 (PMC10882741; doi:10.1186/s12944-024-02045-9)
Supplement: Supplementary file 2 — Supplementary Material 2 [file 12944_2024_2045_MOESM2_ESM.pdf]

EDITORIAL CERTIFICATE

This document certifies that the manuscript below was edited for correct English language usage, grammar, punctuation and spelling by qualified native English speaking editors at Charlesworth Author Services.

Paper Title:

Association of triglyceride glucose-body mass index and hemoglobin glycation index with heart failure prevalence in hypertensive populations: a study across different glucose metabolism status

Author:

Rupeng Wang

Date certificate issued:

February 6, 2024

cwauthors.com
