# Supplementary material for: Association of triglyceride glucose-body mass index and hemoglobin glycation index with heart failure prevalence in hypertensive populations: a study across different glucose metabolism status
Source: Lipids Health Dis. 2024 Feb 22;23:53. doi: 10.1186/s12944-024-02045-9 (PMC10882741; doi:10.1186/s12944-024-02045-9)
Supplement: Supplementary file 3 — Supplementary Material 3 [file 12944_2024_2045_MOESM3_ESM.pdf]

20240208102946952916175150280704

WORD COUNT 4280
TIME SUBMITTED 08-FEB-2024 10:29AM
PAPER ID 106754728

Association of triglyceride glucose-body mass index and hemoglobin glycation index with heart failure prevalence in hypertensive populations: a study across different glucose metabolism status

Abstract

Background: The Triglyceride glucose-body mass index (TyG-BMI) and hemoglobin glycation index (HGI) are well-established surrogate markers for insulin resistance. Nevertheless, the extent to which these markers offer additive predictive value for heart failure (HF) prevalence in hypertensive populations, and their predictive utility across various diabetic statuses, remains to be clarified. Consequently, this study aimed to explore the independent and synergistic effects of TyG-BMI and HGI on HF risk among individuals with different diabetic statuses.

Methods: Data from the study population (n = 9,847) were obtained from the National Health and Nutrition Examination Survey (NHANES). Multivariable logistic regression models were employed to estimate odds ratios (ORs) and 95% confidence intervals (CIs) to assess the combined associations between TyG-BMI and HGI and the prevalence of HF across various diabetic statuses.

Results: In the total population, compared to the reference group (low TyG-BMI and low HGI), the OR (95% CI) for HF prevalence was 1.30 (1.04, 1.64) for the combination of low TyG-BMI and high HGI, 2.40 (1.76, 3.29) for high TyG-BMI and low HGI, and 3.47 (2.41, 4.99) for high TyG-BMI and high HGI. Interestingly, among normoglycemic individuals, higher TyG-BMI and HGI did not significantly increase the prevalence of HF. Conversely, in the prediabetic population, the OR (95%CI) for HF prevalence was 2.42 (1.69, 3.48) for the combination of high TyG-BMI and low HGI, and 4.30 (2.45, 7.54) for high TyG-BMI and high HGI. Similarly, in the diabetic population, the OR (95%CI) for HF prevalence was 2.22 (1.43, 3.45) for low TyG-BMI and high HGI, 4.04 (2.43, 6.73) for high TyG-BMI and low HGI, and 4.13 (2.25, 7.59) for high TyG-BMI and high HGI, compared to low TyG-BMI and

low HGI.

Conclusion: This study reveals that elevated TyG-BMI and HGI levels exert a synergistic impact on the prevalence of HF in hypertensive adults, especially in those with prediabetes and diabetes. Additionally, the presence of prediabetes and diabetes may amplify the detrimental combined effect of TyG-BMI and HGI on HF prevalence.

Keywords: Heart failure, Triglyceride glucose-body mass index, Hemoglobin glycation index, Diabetic status, Hypertension

Background

Heart failure (HF) is a prevalent and serious cardiovascular condition associated with significant morbidity and mortality worldwide[1]. Early identification of individuals at an high risk for HF is crucial for timely interventions and the development of effective preventive strategies[2]. Hypertensive adults with prediabetes and diabetes form a vulnerable population that experiences an increased risk of HF due to the complex interplay of various shared mechanisms, such as insulin resistance (IR), cardiac remodeling and fibrosis, inflammation, oxidative stress, vascular complications, and other common risk factors[3-5].

Recently, there has been a growing interest in novel indices that integrate multiple metabolic parameters to comprehensively assess cardiometabolic risk and predict adverse cardiovascular outcomes. Notably, the triglyceride glucose-body mass index (TyG-BMI) and the hemoglobin glycation index (HGI) stand out in this context[6, 7]. The TyG-BMI, a composite marker, is formulated through the integration of measurements of insulin resistance (TyG) and BMI as a measure of adiposity[6, 8]. Research has demonstrated that elevated levels of TyG-BMI have been associated with an increased risk of various cardiometabolic abnormalities, including IR, diabetes, dyslipidemia, obesity, and cardiovascular disease (CVD)[9-11]. Concurrently, the HGI offers insights into the extent of glycosylated hemoglobin (HbA1c) exceeding the levels anticipated based on glucose levels[12]. It has been proposed as an indicator of glycemic variability and has shown associations with the risk of IR, complications related to diabetes, and CVD[13-15].

Previous research has examined these indices in isolation, frequently neglecting their collective influence, especially in hypertensive adults with prediabetes and diabetes. This oversight is significant, as these populations are particularly vulnerable to HF due to interconnected metabolic, inflammatory,

and cardiovascular processes[16]. The study addresses this by assessing how the interplay between IR (TyG-BMI) and glycemic control variability (HGI) contributes to HF risk, thus providing a more comprehensive risk assessment tool for these high-risk groups[17, 18]. This approach not only fills a critical gap in understanding the multifactorial nature of HF but also aids in the development of targeted preventive and management strategies for HF in hypertensive individuals with varying diabetic statuses. Moreover, the onset and progression of HF are influenced by various factors, including IR, lipid metabolism, and adiposity, which are recognized as primary pathophysiological mechanisms[19, 20]. Therefore, investigating the combined effect of TyG-BMI and HGI on HF can provide valuable insights into their synergistic effects on the development of HF and assist in identifying individuals at the highest risk.

The objective of this study is to examine the combined effect of TyG-BMI and HGI on the prevalence of HF in hypertensive adults with prediabetes and diabetes. By assessing these biomarkers, the goal is to enhance risk stratification and facilitate the development of personalized preventive and management strategies for HF in this vulnerable population.

Methods

Study population

This study utilized data obtained from the National Health and Nutrition Examination Survey (NHANES), a comprehensive program conducted by the Centers for Disease Control and Prevention (CDC) and the National Centers for Health Statistics (NCHS) in the United States. NHANES is designed to assess the health and nutritional status of the U.S. population, following the STROBE guidelines for reporting observational studies. The NHANES study protocol was sanctioned by the NCHS Research Ethics Review Board, and all participants provided written informed consent. Our

study extracted data from the NHANES website (https://www.cdc.gov/nchs/nhanes/index.htm), which encompassed ten survey cycles conducted between 1999 and 2018. After excluding individuals with incomplete data, the final study population comprised 9,847 adults aged 20 years or older who had been diagnosed with hypertension and had participated in NHANES across various survey cycles: 1999-2000 (n = 867), 2001-2002 (n = 943), 2003-2004 (n = 897), 2005-2006 (n = 816), 2007-2008 (n = 1077), 2009-2010 (n = 1102), 2011-2012 (n = 1006), 2013-2014 (n = 1085), 2015-2016 (n = 972), and 2017-2018 (n = 1082). The analysis focused on NHANES participants with hypertension, defined as having a systolic blood pressure of 140 mmHg or higher and/or a diastolic blood pressure of 90 mmHg or higher, taking antihypertensive medications, or having a self-reported history of hypertension[21]. **Fig. 1** presents a flowchart illustrating the selection process of the study population.

Data collection and definitions

This study utilized a comprehensive set of covariates encompassing demographic information, medical history, and laboratory tests to comprehensively explore the research question. Demographic data, collected via self-report NHANES questionnaires, such as age, sex, racial/ethnic background, educational attainment, smoking status, and alcohol consumption. The calculation of BMI involved utilizing participants' weight and height measurements. Information reported by the participants was utilized to identify individuals with physician-diagnosed coronary heart disease (CHD), HF, as well as the use of hypoglycemic drugs, lipid-lowering drugs, and antihypertensive drugs. Fasting venous blood samples were collected on-site during the survey and then sent to the Lipoprotein Analytical Laboratory at Johns Hopkins University School of Medicine. Lipid parameters, including total cholesterol (TC), triglycerides (TG), low-density lipoprotein cholesterol (LDL-C), and high-density lipoprotein cholesterol (HDL-C) concentrations, were measured using the Hitachi 704 Analyzer.

Fasting blood glucose (FBG) concentration was determined using a complete blood count (CBC) identification procedure. HbA1c levels, which provide a measure of long-term blood glucose control, were measured using high-performance liquid chromatography (HPLC) in a centralized laboratory. Hemoglobin concentrations were determined by analyzing whole blood with the Beckman Coulter MAXM instrument using a five-part differential complete blood count method. Albumin concentration was evaluated using specific antibodies that interact with albumin through an immunoturbidimetric assay. The estimated glomerular filtration rate (eGFR) was determined by applying the simplified Modification of Diet in Renal Disease (MDRD) formula, which yields an estimate of kidney function[22].

The participants were classified into three groups based on their diabetic status using the criteria provided by the American Diabetes Association (ADA): normoglycemia, which included individuals with both FBG levels below 5.6 mmol/L and HbA1c levels below 5.7%, and no prescription of hypoglycemic drugs; prediabetes, which was defined by FBG levels ranging from 5.6 to 6.9 mmol/L or HbA1c levels between 5.7 and 6.4%, or both, and no prescription of hypoglycemic drugs. Diabetes was defined as FBG levels equal to or greater than 7.0 mmol/L or HbA1c levels equal to or greater than 6.5%, or both. Additionally, individuals with a prescription of hypoglycemic drugs were classified as diabetic, irrespective of their FBG or HbA1c values[23].

The TyG-BMI index was calculated using the formula: ln [TG (mg/dL) × FBG (mg/dL)/2] × BMI[6]. The HGI value was determined by subtracting the predicted HbA1c value from the observed HbA1c value[7]. The predicted HbA1c in this study was calculated using a correlation regression equation based on the FBG and HbA1c values measured at baseline: Predicted HbA1c level = 3.212 + 0.431 × FBG (mmol/L) ($r = 0.826$; $P < 0.001$), as illustrated in **Fig. 2**.

Ascertainment of outcome

The occurrence of HF events in NHANES was determined using a combination of self-reported data and clinical assessments. NHANES incorporates a comprehensive questionnaire component that inquires about participants' medical history, including prior HF diagnoses. Participants are explicitly queried regarding whether a healthcare professional has ever diagnosed them with HF. Upon confirmation of the initial HF diagnosis, NHANES performs comprehensive medical examinations encompassing various assessments associated with cardiovascular health. Additionally, NHANES may obtain participants' medical records with their consent. This integrative approach, combining self-reported data, clinical assessments, and the review of medical records, aims to enhance the accuracy and validity of HF determination in NHANES.

Statistical analysis

This study adhered to the statistical analysis guidelines provided by the CDC, which can be accessed at https://wwwn.cdc.gov/nchs/nhanes/tutorials/default.aspx. Given the complex multistage stratified probability survey design employed in NHANES, our statistical analysis incorporated sample weights, clustering, and stratification to account for these factors. The baseline characteristics of the study population were presented according to their diabetes status (normoglycemia, prediabetes, diabetes) and HF status (with HF, without HF). Survey-weighted means or medians were employed to report continuous variables, whereas survey-weighted percentages were utilized for categorical variables. Receiver operating characteristic (ROC) analysis was utilized to identify the optimal thresholds of TyG-BMI and HGI for detecting HF prevalence in the total population and across different diabetes statuses. Multivariable logistic regression models were used to estimate odds ratios (ORs) and their corresponding 95% confidence intervals (CIs) to assess the independent and combined associations of

TyG-BMI and HGI with HF prevalence, both for the total population and within specific diabetic subgroups. The selection of adjustment confounders was based on clinical relevance, candidate variables with a *P*-value of <0.05 in univariate analysis, and the availability of events[24]. The statistical analyses were performed using R version 4.0.2 and SPSS (IBM) version 23. Statistical significance was determined based on a two-tailed *P*-value below 0.05.

Results

Baseline characteristics

A total of 9,847 adults with hypertension were included in this study, with a mean age of 58 years. Among the participants, 51.4% were male, 32.3% had normoglycemia, 51.7% had prediabetes, 16% had diabetes, and 5.4% had HF. The baseline characteristics of the study population are presented in **Tables 1** and **Tables 2**, reflecting the participants' diabetes and HF statuses, respectively. **Tables 1** demonstrates that patients with prediabetes and diabetes were older, more likely to be male, of Hispanic ethnicity, less educated, and had a higher prevalence of obesity. Additionally, they exhibited elevated levels of TG, FBG, TyG-BMI, HbA1c, predictive HbA1c, and HGI, as well as lower levels of HDL-C, LDL-C, albumin, and eGFR. These patients also had higher rates of hypoglycemic drugs, lipid-lowering drugs, and antihypertensive drugs, along with a higher prevalence of CHD and HF, compared to those with normoglycemia (P <0.001). Furthermore, the hypertensive population with HF was characterized by older age, lower educational attainment, higher TG, FBG, TyG, TyG-BMI, HbA1c, predictive HbA1c, and HGI levels, as well as lower TC, HDL-C, LDL-C, hemoglobin, albumin, and eGFR levels, and a higher prevalence of hypoglycemic drugs, lipid-lowering drugs, antihypertensive drugs, CHD, and diabetes when compared to individuals without HF (P <0.001) (**Table 2**).

Determination of the optimal thresholds for TyG-BMI and HGI to detect HF

Additional file 1: Table S1 displays the optimal thresholds for TyG-BMI and HGI as determined by ROC analysis. In the total population, the optimal thresholds for detecting TyG-BMI and HGI for HF were 342.89 and 0.25, respectively. Among hypertensive subjects with normoglycemia, the optimal thresholds for TyG-BMI and HGI were determined as 194.27 and -0.19, respectively. In the prediabetic group, the optimal threshold values for TyG-BMI and HGI were 326.08 and 0.47, respectively. In the diabetic group, the optimal threshold values for TyG-BMI and HGI were 342.88 and 0.41, respectively.

Association between TyG-BMI and HGI and the prevalence of HF

The multivariate adjusted model presented in **Table 3** depicts the respective associations of TyG-BMI and HGI with the prevalence of HF. In the general population, individuals with high TyG-BMI exhibited an elevated prevalence of HF (OR, 2.50; 95%CI, 1.95-3.20). Similarly, an increased prevalence of HF was observed with high HGI (OR, 1.33; 95%CI, 1.08-1.63). Among individuals in the normoglycemic group, elevated TyG-BMI and HGI did not significantly increase the prevalence of HF when compared to those with low TyG-BMI or HGI levels. Among the prediabetic population, individuals with high TyG-BMI (OR, 2.60; 95%CI, 1.89-3.58) and high HGI (OR, 1.59; 95%CI, 1.15-2.20) exhibited an elevated prevalence of HF. Likewise, diabetic patients with high TyG-BMI (OR, 2.76; 95%CI, 1.86-4.08) and high HGI (OR, 1.61; 95%CI, 1.14-2.28) displayed an increased prevalence of HF.

Combined association of TyG-BMI and HGI with the prevalence of HF

The multivariate-adjusted model presented in **Table 4** illustrates the combined association of TyG-BMI and HGI with the prevalence of HF. In the joint analysis, using low TyG-BMI and low HGI as the reference, the OR and corresponding 95% CI for the combination of low TyG-BMI and high HGI, high TyG-BMI and low HGI, and high TyG-BMI and high HGI in the total population were 1.30 (1.04,

1.64), 2.40 (1.76, 3.29), and 3.47 (2.41, 4.99), respectively. Furthermore, there was a gradual increase in the prevalence of HF across all four groups, as demonstrated in **Fig. 3A** (*P* for trend < 0.001). In the normoglycemic population, higher TyG-BMI and HGI, as well as their combination, did not elevate the prevalence of HF, as depicted in **Table 4** and **Fig. 3B**. However, among individuals with prediabetes, compared to the combination of low TyG-BMI and low HGI, a higher prevalence of HF was observed in the combination of high TyG-BMI and low HGI (OR, 2.42; 95% CI, 1.69-3.48) and the combination of high TyG-BMI and high HGI (OR, 4.30; 95% CI, 2.45-7.54) (**Fig. 3C**, *P* for trend < 0.001). Similarly, in the diabetic population, the combination of low TyG-BMI and high HGI, high TyG-BMI and low HGI, and high TyG-BMI and high HGI yielded ORs (95% CI) of 2.22 (1.43, 3.45), 4.04 (2.43, 6.73), and 4.13 (2.25, 7.59), respectively, in comparison to the combination of low TyG-BMI and low HGI. Notably, a significant trend towards an increased prevalence of HF was also observed in the combination group among the diabetic population (**Fig. 3D**, *P* for trend < 0.001).

Discussion

This study is the first to investigate the combined association of TyG-BMI and HGI with the prevalence of HF in a US representative hypertensive population, stratified based on diabetes status. The main findings are as follows: 1. Prediabetes and diabetes were characterized by unfavorable baseline factors, such as advanced age, a higher prevalence of obesity, and adverse metabolic profiles; 2. In the overall population, individuals with high TyG-BMI or high HGI had an increased prevalence of HF, with their combination demonstrating the highest prevalence; 3. Among normoglycemic populations, individuals with high TyG-BMI or high HGI did not show an increased prevalence of HF, and their combination did not result in cumulative risk effects; 4. Prediabetic and diabetic individuals with high TyG-BMI or high HGI were at a greater prevalence of HF. In the joint analysis, the combination of high TyG-BMI

and high HGI was significantly associated with a higher prevalence of HF compared to other combinations.

Kiaw Er et al.[6] initially proposed the TyG-BMI and conducted a comparative analysis of TyG-BMI, TyG, and lipid-related parameters in a non-diabetic population, revealing that TyG-BMI exhibited superior diagnostic capabilities for IR. Subsequent studies have demonstrated a positive correlation between elevated TyG-BMI levels and an increased risk of various conditions, including prediabetes, hypertension, hyperuricemia, metabolic syndrome, nonalcoholic liver disease, stroke, and cardiovascular events[9, 11, 25-27]. Nevertheless, limited research has examined the impact of TyG-BMI on occurrence of HF among hypertensive individuals with diabetes and prediabetes. The study suggests that hypertensive individuals with elevated TyG-BMI levels may have a greater likelihood of developing HF, with this association being influenced by the presence of diabetes. Moreover, this connection was observed exclusively in individuals with prediabetes and diabetes. TyG-BMI incorporates multiple factors into a single index, thereby offering a more comprehensive assessment of metabolic health compared to relying solely on individual biomarkers. It captures the interplay among lipid metabolism, glucose homeostasis, and adiposity, all of which play a crucial role in the pathogenesis of HF[28, 29]. Moreover, the practicality, accessibility, and predictive value of TyG-BMI further strengthen its utility in investigating the association with HF and potentially enhancing risk assessment and management strategies in clinical settings.

HbA1c serves as the primary criterion for diagnosing diabetes and prediabetes[30]. However, the HbA1c level measured by the analyzer reflects only 60% to 80% of the average blood glucose level, and the remaining 20% to 40% of HbA1c variation may not be accounted for by the observed HbA1c values[31-33]. To provide a comprehensive reflection of the blood glucose metabolic state, Hempe et al.

proposed HGI, which quantifies interindividual variations in HbA1c resulting from factors beyond blood glucose concentration[34]. While previous studies have established an association between elevated HGI and an increased risk of prediabetes, diabetes, related complications, and CVD, there is limited research exploring the relationship between HGI and the risk of HF[13, 14]. Additionally, since HGI serves as an indicator of blood glucose metabolism variations, its applicability may be influenced by the individual's diabetes status[35]. Hence, it is imperative to evaluate the clinical significance of HGI across various blood glucose states. This study is the first to examine the association between HGI and HF across various diabetic states. The findings indicate that elevated HGI levels are linked to an increased prevalence of HF among individuals with hypertension, although this relationship is influenced by diabetes status. In particular, the positive association between HGI and the prevalence of HF was observed exclusively in individuals with prediabetes and diabetes, while no such association was found in those with normoglycemia. An examination of the relationship between HGI and HF across different diabetic statuses enables clinicians to enhance their understanding of the cardiovascular risk profile among patients with diabetes and adapt their management strategies accordingly. HGI offers supplementary information beyond conventional glycemic markers and has the potential to identify individuals in need of intensive monitoring and more aggressive interventions to prevent or manage HF. Nevertheless, additional research is necessary to validate these findings and ascertain the optimal clinical utilization of HGI in the context of managing HF.

TyG-BMI and HGI were selected as combined variables due to several clinically relevant reasons. First, TyG-BMI combines measurements of triglycerides, glucose, and BMI, all of which are established prevalence factors for HF[36]. This compound variable enables a more comprehensive evaluation of metabolic health and IR[37]. Moreover, HGI reflects the variability in glycemic control

and the glycation of hemoglobin[7]. By integrating these variables, researchers intend to encompass multiple dimensions of metabolic dysfunction and assess their influence on the risk of HF. In addition, the findings demonstrated an independent association between elevated TyG-BMI and HGI values and an increased prevalence of HF. The simultaneous consideration of both variables allows clinicians to achieve a more precise assessment of HF risk and customize management strategies accordingly. Furthermore, examining the relationship between TyG-BMI, HGI, and HF across different diabetic statuses facilitates the identification of high-risk subgroups. The findings indicate that individuals in a prediabetic state who display anomalies in both metabolic markers may necessitate intensified monitoring and early intervention to avert the onset of HF. The identification of individuals exhibiting elevated combined TyG-BMI and HGI values empowers healthcare providers to introduce lifestyle modifications, optimize glycemic control, and initiate preventive measures to mitigate the risk of HF. Early intervention plays a vital role in enhancing outcomes and alleviating the burden of HF in individuals with diabetes.

Notably, gender-related differences significantly influence the prognosis of patients with these conditions. Studies, such as the one indicated by Calabrò P et al[38], have explored these differences, highlighting how gender can affect disease manifestation, progression, and response to treatment. This study, which examines the association of TyG-BMI and HGI with HF risk in hypertensive populations, can benefit from acknowledging these differences. Especially, gender may modulate the risk factors and predictive values of these indices, potentially influencing HF risk. Therefore, future research should consider gender as a critical variable in understanding and managing cardiovascular and metabolic diseases.

3

The biological mechanisms underlying the independent and combined associations of TyG-BMI and

HGI with the prevalence of HF, as well as the modifying effect of diabetes status, are complex and not fully understood. However, there are several plausible explanations. First, TyG-BMI, which combines measures of triglycerides, glucose, and BMI, is considered a marker of IR and metabolic dysfunction[6]. IR, characterized by impaired insulin action and elevated glucose levels, leads to dysregulated lipid metabolism, inflammation, endothelial dysfunction, and oxidative stress[39, 40]. These mechanisms contribute to the development and progression of HF[41, 42]. Elevated TyG-BMI reflects these underlying metabolic abnormalities and can independently increase the prevalence of HF. Second, HGI, which measures the variability in glycemic control and glycation of hemoglobin, reflects long-term glycemic fluctuations and glycation patterns[7]. Chronic hyperglycemia in prediabetes and diabetes can result in the formation of advanced glycation end products and increased oxidative stress, promoting endothelial dysfunction, inflammation, fibrosis, and cardiac remodeling[43, 44]. These pathological processes can contribute to the development and progression of HF. Higher HGI values, indicating poorer glycemic control and increased glycation, have been associated with an independent prevalence of HF. Third, the combined effecct of TyG-BMI and HGI on HF suggests synergistic interactions between IR, metabolic dysfunction, and glycemic variability. IR and hyperglycemia can potentiate each other's deleterious effects, leading to a more pronounced cardiac damage[45]. This synergy may result from the cumulative impact of dysregulated glucose and lipid metabolism, oxidative stress, chronic inflammation, and endothelial dysfunction[46]. The combined effects of TyG-BMI and HGI may amplify these underlying mechanisms, ultimately increasing the prevalence of HF. Fourth, diabetes status, including prediabetes and established diabetes, can modify the relationship between TyG-BMI, HGI, and HF. In individuals with prediabetes and diabetes, the presence of IR, hyperglycemia, and chronic glycemic fluctuations further exacerbates the risk of HF[47]. The

modifying effect of diabetes status implies that the combined association of TyG-BMI and HGI may be more pronounced in individuals with prediabetes or established diabetes compared to those with normoglycemia. Overall, the explanations provided here are based on the current understanding of IR, metabolic dysfunction, glycemic control, and cardiac pathology. Further studies are needed to unravel the intricate interplay between these factors and to elucidate the underlying mechanisms in greater detail.

Strengths and limitations

To the best of our knowledge, this study is the first to investigate the combined association of TyG-BMI and HGI with the prevalence of HF, stratified by diabetes status. However, several limitations should be noted. First, the cross-sectional design limits the ability to establish a cause-and-effect relationship between TyG-BMI, HGI, and the prevalence of HF. It only allows for the assessment of associations at a single time point, without providing information on temporal relationships or the possibility of reverse causality. Second, without longitudinal data, it is challenging to determine whether TyG-BMI and HGI preceded the development of HF or were a consequence of it. Longitudinal studies are better suited for reexamining temporal relationships. Third, despite a large sample size, the generalizability of the findings may be limited to the US adults with hypertension under study. Fourth, the NHANES study's limitation of only providing fasting glucose data without postprandial glucose. It prevents us from fully differentiating between the prediabetes subcategories based on impaired glucose tolerance (IGT). This finding primarily pertains to the impaired fasting glucose (IFG) aspect of prediabetes, and caution should be exercised when generalizing the results to all prediabetic states. Fifth, despite adjusting for enough confounding variables, the possibility of residual confounding and known CVD effect remains. Unmeasured or inadequately measured factors could still influence the

observed associations. Finally, the study relied on self-reported data for recording outcomes, medical history, lifestyle factors, and medication use. Self-reporting introduces potential recall bias and data collection inaccuracies. However, NHANES employs rigorous quality control measures and standardized protocols to ensure the reliability and accuracy of the collected data.

Conclusions

In a nationally representative cohort of hypertensive US adults, the elevated TyG-BMI and HGI levels were associated with an increased prevalence of HF. Notably, the concurrent elevation of TyG-BMI and HGI levels was associated with the highest prevalence of HF when compared to other combinations. The relationships between TyG-BMI and HGI and the prevalences of HF were found to be influenced by the presence of diabetes. Specifically, the adverse effects of TyG-BMI and HGI on HF prevalence were observed solely in populations with prediabetes and diabetes. The clinical relevance of this study lies in its demonstration that in hypertensive patients, particularly those with prediabetes and diabetes, the combined assessment of TyG-BMI and HGI can serve as a significant predictive tool for HF risk, thereby guiding more personalized and effective risk stratification and management strategies in clinical practice.

20240208102946952916175150280704

ORIGINALITY REPORT

9%
SIMILARITY INDEX

PRIMARY SOURCES

1	www.ncbi.nlm.nih.gov<br>Internet	61 words — 1%
2	cardiab.biomedcentral.com<br>Internet	60 words — 1%
3	www.frontiersin.org<br>Internet	57 words — 1%
4	translational-medicine.biomedcentral.com<br>Internet	52 words — 1%
5	worldwidescience.org<br>Internet	51 words — 1%
6	www.jstage.jst.go.jp<br>Internet	51 words — 1%
7	bmcmedicine.biomedcentral.com<br>Internet	20 words — < 1%
8	www.researchsquare.com<br>Internet	19 words — < 1%
9	www.science.gov<br>Internet	19 words — < 1%
10	lipidworld.biomedcentral.com<br>Internet	

19 words — < 1%

11 journals.plos.org
Internet
19 words — < 1%

12 link.springer.com
Internet
17 words — < 1%

13 www.eurekalert.org
Internet
17 words — < 1%

14 www.acnut.com
Internet
16 words — < 1%

15 www.bioseek.eu
Internet
16 words — < 1%

16 Eleanor Mann, A Toby Prevost, Simon Griffin, Ian Kellar et al. "Impact of an informed choice invitation on uptake of screening for diabetes in primary care (DICISION): trial protocol", BMC Public Health, 2009
Crossref
15 words — < 1%

17 kjim.org
Internet
15 words — < 1%

18 www.hindawi.com
Internet
14 words — < 1%

19 Keke Dang, Xuanyang Wang, Jinxia Hu, Yuntao Zhang, Licheng Cheng, Xiang Qi, Lin Liu, Zhu Ming, Xinmiao Tao, Ying Li. "The association between triglyceride-glucose index and its combination with obesity indicators and cardiovascular disease: NHANES 2003–2018", Cardiovascular Diabetology, 2024
Crossref
11 words — < 1%

20 www.omicsdi.org
Internet
11 words — < 1%

21 www.scilit.net
Internet
11 words — < 1%

22 bmccardiovascdisord.biomedcentral.com
Internet
10 words — < 1%

23 medical-data-models.org
Internet
9 words — < 1%

24 www.mdpi.com
Internet
9 words — < 1%

25 www.medrxiv.org
Internet
9 words — < 1%

26 Mohammed N Salman, Thekra Abid Jaber Al-Kashwan, Abdulhussein Faraj Alwan aljanabi. "Exploring the Association between Genetic variant rs5925 in LDLR Gene and the Incidence of T2DM with CAD in Iraqi Population. A case-control Study", Research Square Platform LLC, 2023
Crossref Posted Content
8 words — < 1%

27 P., Spoorthy. "Comparative Study of Serum Triglyceride Level in Normal Pregnancy and in Pregnancy with Hypertensive Disorder and its Maternal and Foetal Outcome", Rajiv Gandhi University of Health Sciences (India), 2023
ProQuest
8 words — < 1%

28 Wanlu Su, Jie Wang, Kang Chen, Wenhua Yan, Zhengnan Gao, Xuelei Tang, Qin Wan, Zuojie Luo, Guang Ning, Yiming Mu. "A higher TyG index level is more likely
8 words — < 1%

to have enhanced incidence of T2DM and HTN comorbidity in elderly Chinese people: a prospective observational study from the Reaction Study", Research Square Platform LLC, 2023
Crossref Posted Content

29 academic.oup.com
Internet
8 words — < 1%

30 dmsjournal.biomedcentral.com
Internet
8 words — < 1%

EXCLUDE QUOTES	OFF	EXCLUDE SOURCES	OFF
EXCLUDE BIBLIOGRAPHY	ON	EXCLUDE MATCHES	OFF
